# ER stress increases expression of intracellular calcium channel RyR1 to modify Ca^2+^ homeostasis in pancreatic beta cells

**DOI:** 10.1016/j.jbc.2023.105065

**Published:** 2023-07-17

**Authors:** Irina X. Zhang, Andrea Herrmann, Juan Leon, Sivakumar Jeyarajan, Anoop Arunagiri, Peter Arvan, Patrick Gilon, Leslie S. Satin

**Affiliations:** 1 Department of Pharmacology and Brehm Diabetes Research Center, University of Michigan Medical School, Ann Arbor, Michigan, USA; 2 J. Crayton Pruitt Family Department of Biomedical Engineering, University of Florida, Gainesville, Florida, USA; 3 Department of Metabolism, Endocrinology & Diabetes, University of Michigan, Ann Arbor, Michigan, USA; 4 Pole of Endocrinology, Diabetes and Nutrition (EDIN), Institute of Experimental and Clinical Research (IREC), Université Catholique de Louvain, Brussels, Belgium

**Keywords:** calcium channels, ryanodine receptor (RyRs), inositol 1,4,5-triphosphate (IP_3_) receptor (IP_3_Rs), endoplasmic reticulum stress (ER stress), beta cells

## Abstract

Pancreatic beta cells maintain glucose homeostasis by secreting pulses of insulin in response to a rise in plasma glucose. Pulsatile insulin secretion occurs as a result of glucose-induced oscillations in beta-cell cytosolic Ca^2+^. The endoplasmic reticulum (ER) helps regulate beta-cell cytosolic Ca^2+^, and ER stress can lead to ER Ca^2+^ reduction, beta-cell dysfunction, and an increased risk of type 2 diabetes. However, the mechanistic effects of ER stress on individual calcium channels are not well understood. To determine the effects of tunicamycin-induced ER stress on ER inositol 1,4,5-triphosphate receptors (IP3Rs) and ryanodine receptors (RyRs) and their involvement in subsequent Ca^2+^ dysregulation, we treated INS-1 832/13 cells and primary mouse islets with ER stress inducer tunicamycin (TM). We showed TM treatment increased *RyR1* mRNA without affecting *RyR2* mRNA and decreased both *IP3R1* and *IP3R3* mRNA. Furthermore, we found stress reduced ER Ca^2+^ levels, triggered oscillations in cytosolic Ca^2+^ under subthreshold glucose conditions, and increased apoptosis and that these changes were prevented by cotreatment with the RyR1 inhibitor dantrolene. In addition, we demonstrated silencing RyR1-suppressed TM-induced subthreshold cytosolic Ca^2+^ oscillations, but silencing RyR2 did not affect these oscillations. In contrast, inhibiting IP3Rs with xestospongin-C failed to suppress the TM-induced cytosolic Ca^2+^ oscillations and did not protect beta cells from TM-induced apoptosis although xestospongin-C inclusion did prevent ER Ca^2+^ reduction. Taken together, these results show changes in RyR1 play a critical role in ER stress-induced Ca^2+^ dysfunction and beta-cell apoptosis.

Calcium (Ca^2+^) is an essential cellular signal. In pancreatic beta cells, Ca^2+^ triggers insulin secretion to maintain postprandial blood glucose (1). Ca^2+^ is sequestered within the endoplasmic reticulum (ER), the organelle where the synthesis and folding of secretory proteins occurs, along with lipid synthesis (2, 3). A well-functioning ER is critical for proper beta-cell function and survival (4, 5, 6, 7). On the other hand, ER malfunction can potentially lead to type 2 diabetes, and there is evidence that the unfolded protein response (UPR) is activated in islets from type 2 diabetes patients or animal models of diabetes (6, 8).

Decreased ER Ca^2+^ concentration ([Ca^2+^]_ER_) is associated with ER stress and apoptosis and accompanies many pathologies (6, 9, 10). ER Ca^2+^ is regulated by a balance between Ca^2+^ uptake *via* sarco/endoplasmic reticulum Ca^2+^-ATPases (SERCA pumps) and Ca^2+^ release by inositol 1,4,5-trisphosphate receptors (IP3Rs) and ryanodine receptors (RyRs) (11). Both IP3Rs and RyRs are gated by Ca^2+^, and both trigger Ca^2+^-induced Ca^2+^ release under certain conditions (12, 13, 14, 15). There are three known isoforms of IP3Rs: IP3R1, IP3R2, and IP3R3 (16); IP3R1 is the most abundant isoform in beta cells (16). RyRs are also encoded by three separate genes, *RyR1*, *RyR2*, and *RyR3* (14, 17), with RyR2 being the most abundant one in beta cells (18). Beta-cell IP3Rs have been extensively studied, and their role in G-protein coupled receptor-coupled intracellular Ca^2+^ release is well established (14, 15). In contrast, the role of RyRs has been more limited and controversial, in part because RyR expression in beta cells is very low (18).

In this study, we tested whether ER stress differentially regulated RyRs and IP3Rs in beta cells to better define their respective roles in beta-cell function, such as in ER Ca^2+^ handling and the production of cytosolic Ca^2+^ ([Ca^2+^]_cyto_) oscillations. We used tunicamycin (TM) to experimentally induce ER stress in the rat insulin-secreting INS-1 832/13 beta-cell line, as well as isolated mouse islets. TM activates the UPR by inhibiting N-acetylglucosamine phosphotransferase, leading to protein misfolding in the ER (19). TM treatment triggered [Ca^2+^]_cyto_ oscillations and apoptosis through RyR1-mediated ER Ca^2+^ release. IP3Rs in contrast appeared to play a minor role in these processes. Importantly, while RyR2 has been considered the key isoform regulating ER Ca^2+^ in beta cells (17, 18), our results emphasize the importance of RyR1 in ER stress. Ultimately, these findings suggest that it might be beneficial to target RyR1 as a potential therapy to alleviate ER stress-mediated beta-cell dysfunction in type 2 diabetes.

## Results

In previous work, we reported that chemically inducing ER stress in beta cells using TM activated store-operated Ca^2+^ entry (SOCE) and led to the appearance of [Ca^2+^]_cyto_ oscillations in parallel with oscillations in membrane potential (20). The increase in [Ca^2+^]_cyto_ concomitantly augmented insulin secretion under what would normally be subthreshold glucose conditions, for example, in medium containing 5 mM glucose (20). TM induced a reduction in [Ca^2+^]_ER_, which we suggested was likely the proximal trigger for inducing extracellular Ca^2+^ influx *via* SOCE. Ca^2+^ can diffuse out of the ER through open IP3Rs, RyRs, or possibly the ER translocon (not addressed in this paper) (6). In response to ER stress, IP3Rs and RyRs may become dysregulated, resulting in enhanced stimulated release of ER Ca^2+^ (18). As this possibility was not addressed in our previous work, here we decided to investigate the respective roles of IP3Rs and RyRs in altered beta-cell function under ER stress conditions. To do so, we took advantage of the selective ER Ca^2+^ channel antagonists xestospongin-C (XeC, 1 μM) (21) and dantrolene (Dan, 10 μM) (22), to block IP3Rs and RyR1, respectively. Dan mainly selectively inhibits RyR1 over RyR2 (22, 23, 24), but the selectivity remains controversial (25).

**Figure 1:**
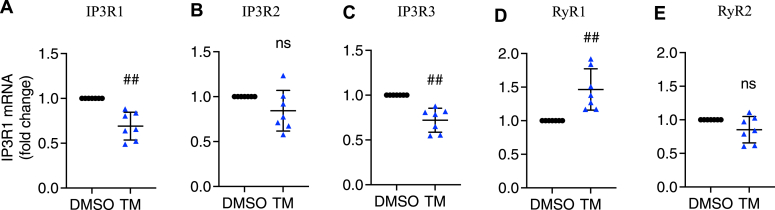
**Tunicamycin altered *IP3R*s and *RyR*s transcripts.** INS-1 832/13 cells were treated with vehicle control (DMSO) or tunicamycin (TM, 10 μg/ml) for 16 h in 11 mM glucose INS-1 832/13 culture medium. *IP3R* isoforms and *RyR* isoforms mRNA levels were measured in INS-1 832/13 cells. *A*, *IP3R1*. *B*, *IP3R2*. *C*, *IP3R3*. *D*, *RyR1*. *E*, *RyR2*. All values shown are means ± SD; ##, *p* < 0.01, ns = not significant; n = 7 independent experiments, by one sample *t* test with hypothetical value set for 1.0.

We first analyzed the effect of TM on the expression of each of the receptor isoforms. As shown in Figure 1, *A*–*C*, TM treatment decreased *IP3R1* and *IP3R3* mRNAs, while the *IP3R2* transcript remained unchanged after a 16 h exposure to TM in INS-1 832/13 cells. We also found that *RyR1* mRNA was increased by TM treatment, while the level of *RyR2* transcript was unchanged (Fig. 1, *D* and *E*). While we wished to measure RyRs and IP3Rs at the protein level, our efforts to do so were hampered by the lack of commercially available, working antibodies for these ER Ca^2+^ channel proteins.

**Figure 2:**
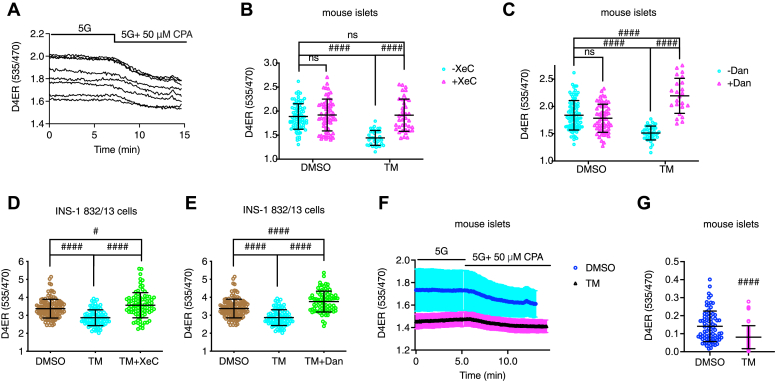
**Xestospongin C or dantrolene prevented tunicamycin-induced basal [Ca**^**2+**^**]**_**ER**_**reduction.** Mouse pancreatic islets or INS-1 832/13 cells were infected with an adenovirus expressing a beta-cell-directed D4ER probe for 3 h or 2 h, respectively, followed by a 48-h recovery period. Islets and INS-1 832/13 cells were then treated with vehicle control (DMSO), tunicamycin (TM, 10 μg/ml), xestospongin C (XeC, 1 μM), dantrolene (Dan, 10 μM), TM+ XeC, or TM+ dantrolene, for 16 h in 11 mM glucose culture medium. *A*, representative basal [Ca^2+^]_ER_ traces for control islets obtained in 5 mM glucose solution before and after cyclopiazonic acid (CPA, 50 μM) application. *B* and *C*, islet [Ca^2+^]_ER_ for indicated conditions in 5 mM glucose solution containing 0.2 mM diazoxide. *D* and *E*, INS-1 832/13 cells [Ca^2+^]_ER_ for indicated conditions in 5 mM glucose solution containing 0.2 mM diazoxide. *F*, representative basal [Ca^2+^]_ER_ traces for control or TM-treated islets obtained in 5 mM glucose solution before and after cyclopiazonic acid (CPA, 50 μM) application. Values shown are means ± SD. *G*, the reduction of [Ca^2+^]_ER_ in response to CPA. Difference = D4ER ratios at the beginning of the recordings before CPA application − D4ER ratios at the end of the recordings (in steady-state) after CPA application for indicated conditions. Each data point shown was a D4ER ratio obtained for one selected region of interest, a single cell or small group of cells. *B*, row factor F(1, 214) = 30.21, *p* < 0.0001, column factor F(1, 214) = 37.83, *p* < 0.0001, interaction F(1, 214) = 29.13, *p* < 0.0001. *C*, row factor F(1, 211) = 1.242, *p* = 0.2663, column factor F(1, 211) = 65.89, *p* < 0.0001, interaction F(1, 211) = 90.98, *p* < 0.0001. All values shown are means ± SD. #, *p* < 0.05, ####, *p* < 0.0001; ns = not significant. *B* and *C*, n = 35 to 90 cells in intact islets isolated from at least three mice, by two-way ANOVA with post hoc multiple comparison by Tukey’s procedure; *D* and *E*, n = 85 to 125 INS-1 832/13 cells from three independent experiments, by one-way ANOVA with post hoc multiple comparison by Tukey’s procedure; *G*, n = 58 to 82 cells in intact islets isolated from five mice, by student’s *t* test.

To test whether blocking IP3Rs or RyRs prevented the TM-mediated reduction of ER Ca^2+^ (20), the ER Ca^2+^ probe D4ER was transiently expressed in islets and INS-1 832/13 cells with adenovirus. Islets and INS-1 832/13 cells were then treated with vehicle control (DMSO), TM, XeC, Dan, TM+XeC, or TM+Dan for 16 h, and [Ca^2+^]_ER_ was measured using a recording solution containing 5 mM glucose. Representative traces show [Ca^2+^]_ER_ (Y-axis, in arbitrary units) in islets and the effect of the reversible SERCA blocker cyclopiazonic acid (CPA, 50 μM) reduced ER Ca^2+^, as expected (26) (Fig. 2*A*). Steady-state [Ca^2+^]_ER_ is measured in 5 mM glucose in islets (Fig. 2, *B* and *C*) and in INS-1 832/13 cells (Fig. 2, *D* and *E*) in the absence of CPA. Exposing islets to TM for 16 h significantly reduced steady-state [Ca^2+^]_ER_ compared to controls, and the presence of either XeC or Dan with TM prevented ER Ca^2+^ loss. Interestingly, including Dan with TM slightly increased [Ca^2+^]_ER_ over control levels but had no effect in the controls, perhaps because ER Ca^2+^ leak through RyRs was low in the controls (Fig. 2*C*). Figure 2*F* shows representative ER Ca^2+^ responses to CPA in 5 mM glucose in control or TM-treated islets, and we saw that TM-treated islets had lower ER Ca^2+^ both before and after CPA acute application compared to control. The change in D4ER arbitrary units was calculated by subtracting the unit in steady state at the end of the recordings after CPA application from the unit at the beginning of the recordings before CPA application. We found that the drop in ER Ca^2+^ was significantly smaller after TM exposure than control (Fig. 2*G*).

**Figure 3:**
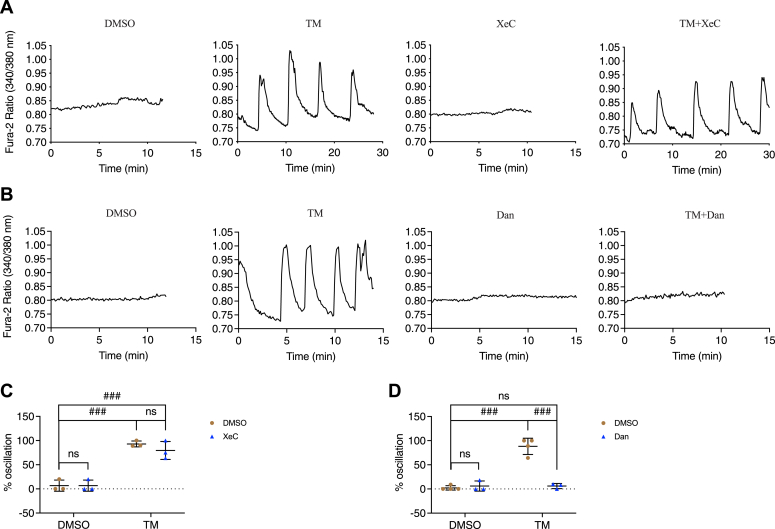
**Differential effects of xestospongin C and dantrolene on [Ca**^**2+**^**]**_**cyto**_**oscillations under subthreshold glucose conditions.** Isolated pancreatic mouse islets were treated with vehicle control (DMSO); tunicamycin (TM, 10 μg/ml); *A* and *C*, xestospongin C (XeC, 1 μM) or TM+XeC; *B* and *D*, dantrolene (Dan, 10 μM) or TM+Dan for 16 h in 11 mM glucose islet culture medium. *A* and *B*, the responses of [Ca^2+^]_cyto_ to the solution containing 5 mM glucose under the indicated conditions. *C* and *D*, percentage of oscillating islets. *C*, row factor F(1, 8) = 116.9, *p* < 0.0001, column factor F(1, 8) = 0.8306, *p* = 0.3887, interaction F(1, 8) = 0.8306, *p* = 0.3887. *D*, row factor F(1, 10) = 53.43, *p* < 0.0001, column factor F(1, 10) = 44.28, *p* < 0.0001, interaction F(1, 10) = 53.31, *p* < 0.0001. All values shown are means ± SD, ###, *p* < 0.005, ns = not significant; n = 3 to 4 mice, by two-way ANOVA with post hoc multiple comparison by Tukey’s procedure.

To determine whether IP3Rs and/or RyRs play a role in the production of the oscillations seen in subthreshold glucose in stressed beta cells, islets were exposed to a vehicle control (DMSO), TM, XeC, Dan, TM+XeC, or TM+Dan for 16 h before recording [Ca^2+^]_cyto_ in a solution containing 5 mM glucose. Mouse islets cultured overnight in media containing 11 mM glucose do not typically exhibit oscillations in [Ca^2+^]_cyto_ when acutely exposed to subthreshold glucose levels (*i.e.*, glucose concentrations <7 mM) (20, 27, 28). As expected, control islets displayed little or no oscillatory [Ca^2+^]_cyto_ (Fig. 3). In contrast, ∼80% of TM-treated islets exhibited Ca^2+^ oscillations in 5 mM glucose (Fig. 3) due to the activation of SOCE, as demonstrated in our previous study, even though these channels are not normally involved in the production of glucose-induced islet oscillations under physiological conditions (20, 29). Including XeC with TM had no effect on the production of Ca^2+^ oscillations in stressed islets (Fig. 3, *A* and *C*); however, TM-triggered oscillations were suppressed when Dan was present (<5% oscillating islets) (Fig. 3, *B* and *D*), suggesting RyRs but not IP_3_Rs were involved in their genesis. In Dan or XeC-treated islets, as for DMSO, little or no oscillatory Ca^2+^ activity was observed in the absence of TM (Fig. 3).

**Figure 4:**
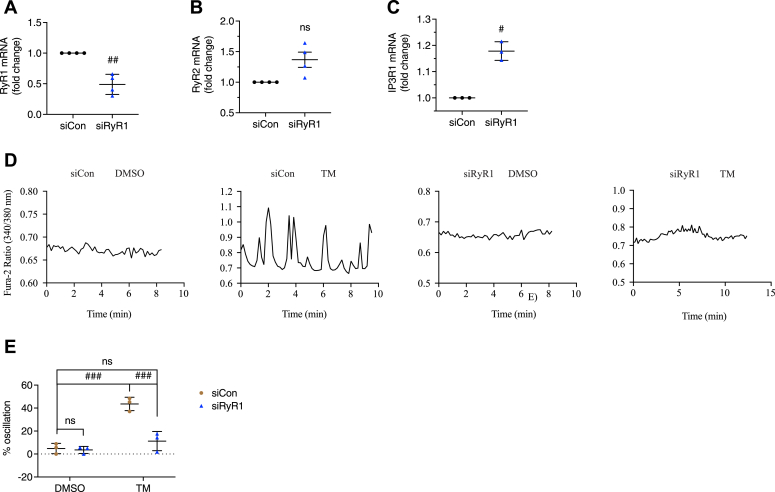
**RyR1-knockdown inhibited tunicamycin-triggered [Ca**^**2+**^**]**_**cyto**_**oscillations.***A*, RyR1-knockdown in INS-1 832/13 cells was assessed by qPCR 64 h after siRNA transfection. *B* and *C*, *RyR2* mRNA (*B*) and *IP3R1* mRNA (*C*) levels in INS-1 832/13 cells after transfection with *RyR1* siRNA. INS-1 832/13 cells were treated with vehicle control (DMSO) or tunicamycin (TM, 10 μg/ml) for 16 h after transfecting with *RyR1* siRNA or negative control siRNA for 48 h. *D*, the responses of [Ca^2+^]_cyto_ to a solution containing 5 mM glucose. *E*, percentage of oscillating INS-1 832/13 cells. All values shown are means ± SD. *A*–*C*: #, *p* < 0.05, ##, *p* < 0.01, ns = not significant; n = 3 to 4 independent experiments, by one sample *t* test with hypothetical value set for 1.0. *E*, row factor F(1, 8) = 48.52, *p* = 0.0001, column factor F(1, 8) = 25.36, *p* = 0.0010, interaction F(1, 8) = 21.72, *p* = 0.0016. ###, *p*< 0.005, ns = not significant; n = 3 independent experiments, by two-way ANOVA with post hoc multiple comparison by Tukey’s procedure.

To confirm that RyR1 played a role in the production of TM-triggered [Ca^2+^]_cyto_ oscillations in 5 mM glucose, we transfected *RyR1* siRNA in INS-1 832/13 cells, resulting in a 50% decrease of *RyR1* mRNA (Fig. 4*A*). Decreasing RyR1 expression may increase *RyR2* mRNA levels, but not significantly (Fig. 4*B*). *IP3R1* mRNA was found to be elevated (Fig. 4*C*). Transfected cells were recorded using imaging buffer containing 5 mM glucose (Fig. 4*D*). After 16 h of TM treatment, the percentage of cells displaying [Ca^2+^]_cyto_ oscillations was decreased in RyR1-knockdown cells (∼10%) compared to siCon cells (∼40%) (Fig. 4*E*). RyR1 knockdown did not affect the Ca^2+^ activity of unstressed control cells (Fig. 4, *D* and *E*).

**Figure 5:**
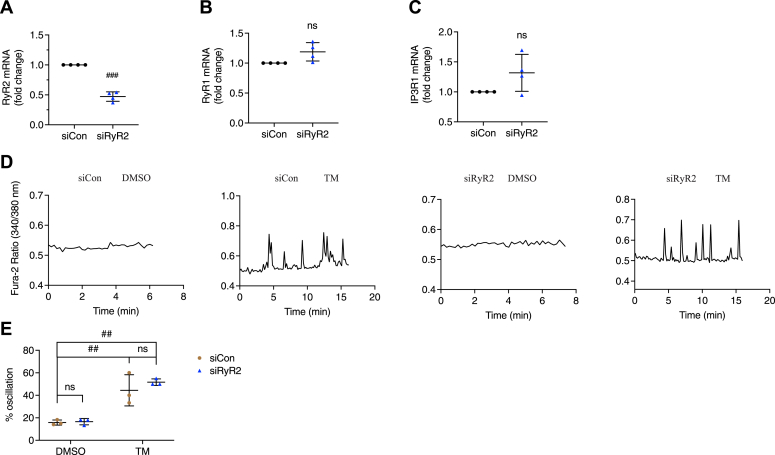
**RyR2-knockdown did not affect tunicamycin-triggered [Ca**^**2+**^**]**_**cyto**_**oscillations.***A*, RyR2-knockdown in INS-1 832/13 cells was assessed by qPCR 64 h after siRNA transfection. *B* and *C*, *RyR1* mRNA (*B*) and *IP3R1* mRNA (*C*) levels in INS-1 832/13 cells after transfection with *RyR2* siRNA. INS-1 832/13 cells were treated with vehicle control (DMSO) or tunicamycin (TM, 10 μg/ml) for 16 h after transfecting with *RyR2* siRNA or negative control siRNA for 48 h. *D*, the responses of [Ca^2+^]_cyto_ to solution containing 5 mM glucose. *E*, percentage of oscillating INS-1 832/13 cells. All values shown are means ± SD. *A*–*C*: ###, *p* < 0.005, ns = not significant; n = 4 independent experiments, by one sample *t* test with hypothetical value set for 1.0. *E*, row factor F(1, 8) = 57.05, *p* < 0.0001, column factor F(1, 8) = 0.8975, *p* = 0.3712, interaction F(1, 8) = 0.5828, *p* = 0.4671. ##, *p* < 0.01; n = 3 independent experiments, by two-way ANOVA with post hoc multiple comparison by Tukey’s procedure.

RyR2, rather than RyR1, is the predominant RyR isoform of beta cell in terms of abundance (18). To study whether RyR2 is also involved in TM-induced [Ca^2+^]_cyto_ oscillations in 5 mM glucose, we silenced RyR2 using siRNA in INS-1 832/13 cells and achieved a ∼50% reduction in *RyR2* mRNA (Fig. 5*A*). Neither *RyR1* or *IP3R1* mRNA was changed after RyR2 knockdown (Fig. 5, *B* and *C*). The cytosolic Ca^2+^ levels of the transfected cells were recorded using imaging buffer containing 5 mM glucose (Fig. 5*D*). The percentage of cells displaying [Ca^2+^]_cyto_ oscillations in response to 16 h of TM treatment in this case was unaffected by silencing RyR2 (Fig. 5*E*).

**Figure 6:**
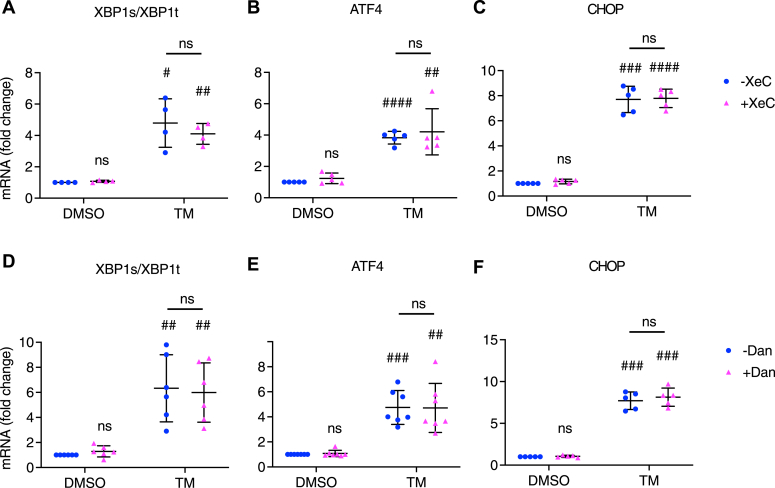
**Xestospongin C or dantrolene did not attenuate UPR activation.** INS-1 832/13 cells were treated with vehicle control (DMSO); tunicamycin (TM, 10 μg/ml); *A*–*C*, xestospongin C (XeC, 1 μM) or TM+XeC; *D*–*F*, dantrolene (Dan, 10 μM) or TM+Dan for 6 h in 11 mM glucose INS-1 832/13 culture medium. Various ER stress markers were measured. *A* and *D*, Spliced *XBP1*/total *XBP1* ratio. *B* and *E*, *ATF4*. *C* and *F*, *CHOP*. All values shown are means ± SD. #, *p* < 0.05; ##, *p* < 0.01; ###, *p* < 0.005, ####, *p* < 0.0001; ns = not significant, compared with control DMSO; n = 4–7 independent experiments, by one sample *t* test with hypothetical value set for 1.0. A comparison between TM-XeC and TM+XeC or TM-Dan and TM+Dan was done by student’s *t* test.

Activation of the UPR occurs in response to TM in many cell types, including beta cells (18, 20, 30). We previously showed that increases in several of the canonical markers of the ER stress response, such as spliced XBP1, CHOP, and BiP, occurred in TM-treated INS-1 832/13 cells and mouse islets (20). To better define the respective roles of IP3Rs and RyRs in TM-induced ER stress in beta cells, INS-1 832/13 cells were treated with TM (10 μg/ml) or vehicle control (DMSO) with or without XeC (1 μM) or Dan (10 μM) for 6 h. Total mRNA was then extracted and quantified, as we had previously observed that spliced *XBP1* reached its peak at this time (20). However, neither XeC nor Dan suppressed UPR activation, as indicated by an increased ratio of spliced *XBP1*/total *XBP1* (Fig. 6, *A* and *D*), *ATF4* (Fig. 6, *B* and *E*), or *CHOP* (Fig. 6, *C* and *F*), which we observed when these inhibitors were included with TM. Thus, blocking IP3Rs or RyR1 failed to prevent UPR activation in TM-treated beta cells.

**Figure 7:**
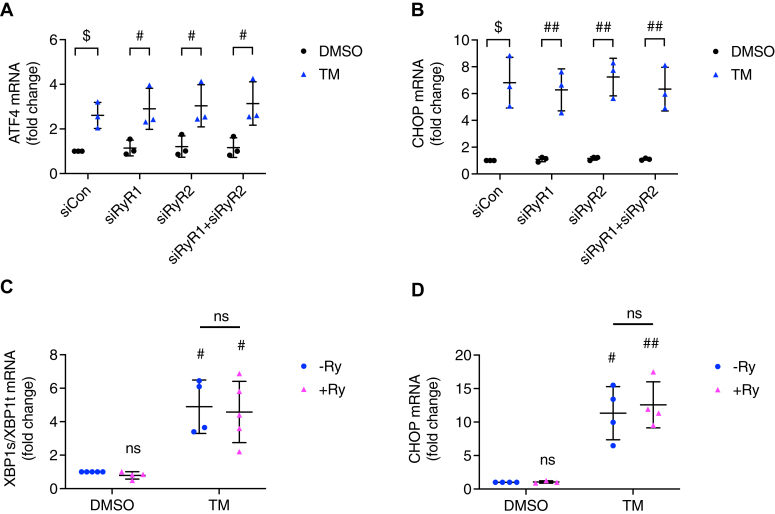
**Inhibiting ryanodine receptors did not affect TM-triggered UPR activation.** INS-1 832/13 cells were transfected with siRNA for control, *RyR1*, *RyR2*, or *RyR1*+*RyR2* for 64 h and treated with vehicle control (DMSO) or TM (10 μg/ml) for 6 h in 11 mM glucose INS-1 832/13 culture medium. *A* and *B*, *ATF4* (*A*) and *CHOP* (*B*) were measured. All values shown are means ± SD. $, *p* < 0.05, compared with control DMSO; n = 3 independent experiments, by one sample *t* test with hypothetical value set for 1.0. #, *p* < 0.05; ##, *p* < 0.01, by student’s *t* test. INS-1 832/13 cells were treated with vehicle control (DMSO), ryanodine (RyR, 100 μM), tunicamycin (TM, 10 μg/ml), or TM+Ry for 6 h in 11 mM glucose INS-1 832/13 culture medium. *C*, spliced *XBP1*/total *XBP1* ratio; *D*, *CHOP* were measured. All values shown are means ± SD. #, *p* < 0.05, ##, *p* < 0.01; ns = not significant; n = 3 to 5 times independent experiments, by one sample *t* test with hypothetical value set for 1.0. Comparison between TM-Ry and TM+Ry was done by student’s *t* test.

To confirm that blocking RyRs does not affect TM-triggered UPR activation, we assessed *ATF4* and *CHOP* expression in RyR1, RyR2, or RyR1+2 knockdown cells and observed that neither *ATF4* nor *CHOP* upregulation was reversed by silencing these isoforms individually or collectively (Fig. 7, *A* and *B*). To further confirm this, we also examined the effect of blocking RyRs on UPR activation using ryanodine (Ry), a classic nonspecific RyRs blocker (at 100 μM, Ry is a RyR blocker, while at nanomolar concentrations, Ry is a RyR activator (31)). As shown in Figure 7, *C* and *D*, when INS-1 832/13 cells were treated with TM for 6 h, neither the ratio of spliced *XBP1*/total *XBP1* nor *CHOP* mRNA were reduced by the inclusion of 100 μM of Ry, which was similar to what we observed upon RyR1+2 knockdown (Fig. 7, *A* and *B*).

**Figure 8:**
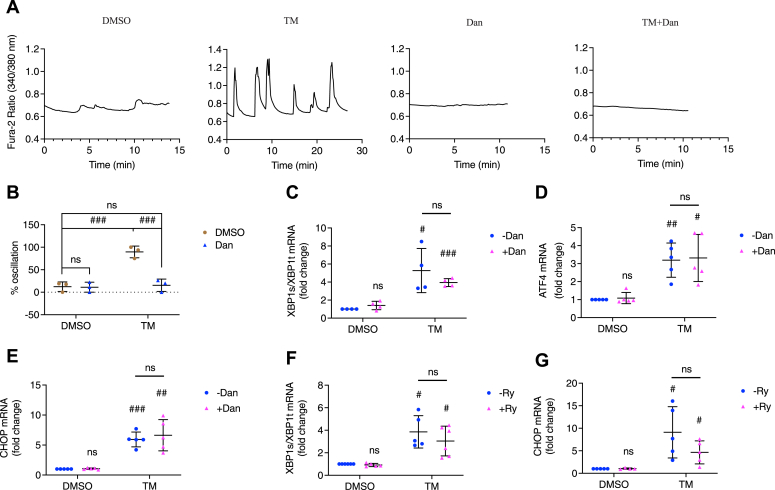
**Subthreshold [Ca**^**2+**^**]**_**cyto**_**oscillations and UPR activation in beta cells stressed with low dose of tunicamycin.** Isolated pancreatic mouse islets were treated with vehicle control (DMSO), tunicamycin (TM, 300 nM), dantrolene (Dan, 10 μM), or TM+Dan for 24 h in 11 mM glucose culture medium. *A*, the responses of [Ca^2+^]_cyto_ to the solution containing 5 mM glucose under the indicated conditions. *B*, percentage of oscillating islets. Row factor F(1, 8) = 33.68, *p* = 0.0004, column factor F(1, 8) = 28.60, *p* = 0.0007, interaction F(1, 8) = 26.92, *p* = 0.0008. All values shown are means ± SD. ###, *p* < 0.005, ns = not significant; n = 3 mice, by two-way ANOVA with post hoc multiple comparison by Tukey’s procedure. INS-1 832/13 cells were treated with vehicle control (DMSO), tunicamycin (TM, 300 nM), dantrolene (Dan, 10 μM), TM+Dan, ryanodine (Ry, 100 μM), or TM+Ry for 6 h in INS-1 832/13 culture medium. *C* and *F*, spliced *XBP1*/total *XBP1* ratio; *D*, *ATF4*; and *E* and *G*, *CHOP* were measured. All values shown are means ± SD. #, *p* < 0.05, ##, *p* < 0.01; ###, *p* < 0.005, ns = not significant; n = 4 to 5 times independent experiments, by one sample *t* test with hypothetical value set for 1.0. Comparison between TM-Dan and TM+Dan or between TM-Ry and TM+Ry was done by student’s *t* test.

We also treated islets with a much lower dose of TM (300 nM) for 24 h and verified if such low dose of TM could trigger UPR activation. We observed that 90% of TM-treated islets exhibited subthreshold Ca^2+^ oscillations, and the inclusion of Dan lowered the percentage to 10% (Fig. 8, *A* and *B*). In addition, we saw increased ratio of spliced *XBP1*/total *XBP1*, *ATF4*, and *CHOP* mRNA after a 6 h treatment that was not affected by Dan (Fig. 8, *C*–*E*) or Ry. Although Ry seemed to suppress TM-induced upregulation of *XBP1s*/*XBP1t* ratio and *CHOP* mRNA, the difference was not statistically significant (Fig. 8, *F* and *G*).

**Figure 9:**
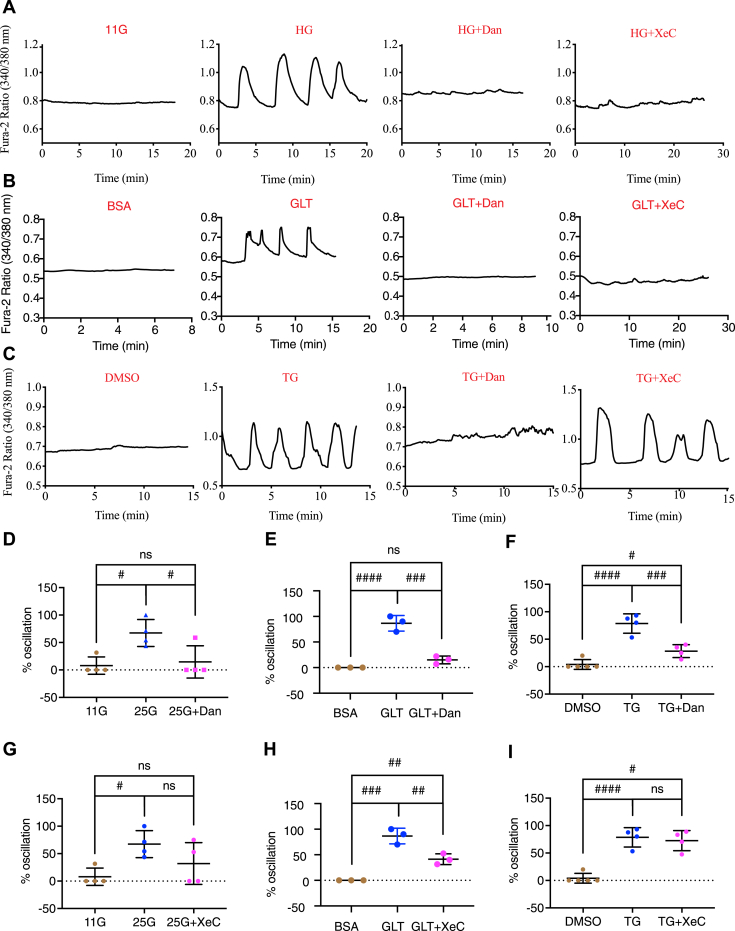
**Differential effects of dantrolene and xestospongin C on [Ca**^**2+**^**]**_**cyto**_**under subthreshold glucose conditions in islets challenged by various ER stress inducers.** Isolated pancreatic mouse islets were cultured in (*A*) control 11 mM glucose, high glucose (HG, 25 mM), (*B*) control BSA (0.92%, 11 mM glucose), GLT (400 μM palmitate precomplexed to 0.92% BSA, 16.7 mM glucose), (*C*) control DMSO or thapsigargin (TG, 200 nM) in the presence or absence of dantrolene (Dan, 10 μM) or xestospongin C (XeC, 1 μM) for 16 h. *A*–*C*, the responses of [Ca^2+^]_cyto_ to solution containing 5 mM glucose under the indicated conditions. *D*–*I*, percentage of oscillating islets. All values shown are means ± SD; #, *p* < 0.05; ##, *p* < 0.01, ###, *p* < 0.005, ####, *p* < 0.0001, ns = not significant; n = 3 to 5 mice, by one-way ANOVA with post hoc multiple comparison by Tukey’s procedure.

We previously reported that culturing mouse islets in high glucose (HG, 25 mM) for 16 h induced subthreshold [Ca^2+^]_cyto_ oscillations (20). We first confirmed that overnight HG treatment induced ER stress evidenced by upregulation of *CHOP* mRNA levels (Fig. S1*A*) in INS-1 832/13 cells. We then measured *RyR* isoforms and *IP3R1* mRNA levels in the cells and observed an upregulation of *RyR1* mRNA after high glucose culturing, although not significant (*p* = 0.0832) (Fig. S1*B*), and unchanged *RyR2* (Fig. S1*C*) or *IP3R1* mRNA levels (Fig. S1*D*). We next tested whether XeC or Dan affected [Ca^2+^]_cyto_ oscillations (Fig. 9*A*). Dan significantly reduced the percentage of islets exhibiting oscillations seen following HG treatment from ∼70% to ∼20% (Fig. 9*D*), and XeC decreased it to ∼30% (average).

Combining high-glucose conditions with elevated free fatty acids to generate glucolipotoxic (GLT) conditions has also been used to mimic the T2D conditions (32), and GLT has been shown to induce ER stress in beta cells (33). We treated mouse islets with BSA control (11 mM glucose) or GLT (400 μM palmitate precomplexed to 0.92% BSA, 16.7 mM glucose) for 16 h in the presence or absence of XeC or Dan (Fig. 9*B*). We found ∼80% oscillation in GLT-treated islets, and Dan suppressed Ca^2+^ oscillation to ∼10% (Fig. 9*E*). XeC also decreased the percentage of oscillation (to ∼40%, Fig. 9*H*), but not as much as Dan.

Thapsigargin (TG) is another widely used ER stress chemical inducer (34). We previously also reported subthreshold [Ca^2+^]_cyto_ oscillations in TG (200 nM, 16 h)-treated mouse islets (20). Here, we found that Dan significantly decreased the percentage of islets exhibiting oscillations in response to TG treatment (from ∼80% to ∼25%) (Fig. 9*F*), while XeC did not show much affect (Fig. 9*I*).

**Figure 10:**
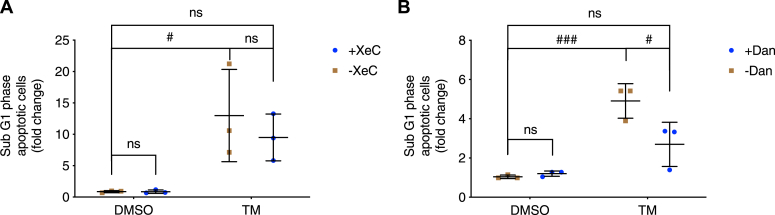
**Differential effects of xestospongin C and dantrolene on beta-cell apoptosis.** INS-1 832/13 cells were treated with vehicle control (DMSO) or tunicamycin (TM, 10 μg/ml), (*A*) xestospongin C (XeC, 1 μM) or TM+XeC; (*B*) dantrolene (Dan, 10 μM) or TM+Dan for 24 h in 11 mM glucose INS-1 832/13 culture medium. Late-stage apoptotic INS-1 832/13 cells are shown using the sub-G1 assay measured by flow cytometry. Fold change was derived by comparing to DMSO group. *A*, row factor F(1, 8) = 19, *p* = 0.0024, column factor F(1, 8) = 0.5346, *p* = 0.4856, interaction F(1, 8) = 0.5338, *p* = 0.4859. *B*, row factor F(1, 8) = 41.53, *p* = 0.0002, column factor F(1, 8) = 6.086, *p* = 0.0389, interaction F(1, 8) = 8.125, *p* = 0.0215. All values shown are means ± SD. #, *p* < 0.05, ###, *p* < 0.005, ns = not significant; n = 3 independent experiments, by two-way ANOVA with post hoc multiple comparison by Tukey’s procedure.

Programmed cell death or apoptosis has been shown to occur in beta cells in response to prolonged ER stress (6, 20, 34, 35). We examined how XeC or Dan affected the percentage of cells in the sub-G1 phase of the cell cycle, a measure of cell entry into late-stage apoptosis (36). As expected at 24 h, TM significantly increased the percentage of INS-1 832/13 cells undergoing apoptosis compared to DMSO- or Dan/XeC-treated controls (Fig. 10). XeC was without significant prevention on TM-induced cell apoptosis, but Dan+TM considerably reduced apoptosis to near-control levels, suggesting a potential linkage between RyR1 and TM-triggered beta-cell apoptosis.

**Figure 11:**
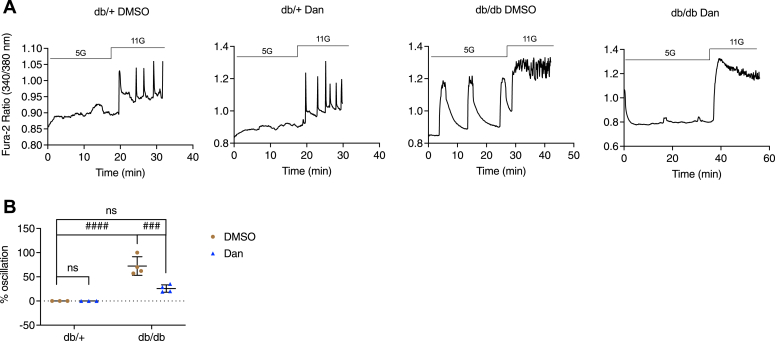
**Dantrolene suppressed subthreshold [Ca**^**2+**^**]**_**cyto**_**oscillation in islets from *db/db* mice.** Pancreatic islets were isolated from female *db/+* and *db/db* mice (5–7 weeks old) and treated with control DMSO or dantrolene (Dan, 10 μM) for 16 h in 11 mM glucose islet culture medium. *A*, the responses of [Ca^2+^]_cyto_ to the solution containing 5 mM and 11 mM glucose under the indicated conditions. *B*, percentage of oscillating islets. Row factor F(1, 10) = 64.45, *p* < 0.0001, column factor F(1, 10) = 14.51, *p* = 0.0034, interaction F(1, 10) = 14.51, *p* = 0.0034. All values shown are means ± SD; ###, *p* < 0.005; ####, *p* < 0.0001, ns = not significant; n = 3 to 4 mice, by two-way ANOVA with post hoc multiple comparison by Tukey’s procedure.

To test the possible relevance of these results to diabetes, we extended our study to islets isolated from *db/db* mice, an animal model of type 2 diabetes. These mice harbor a mutation in the leptin receptor and have been shown to exhibit a left shift in their glucose sensitivity over time (37) and upregulation of ER stress markers BiP, p58, CHOP, Atf3, and Grp94 at the mRNA levels in 5- to 8-week-old *db/db* mice compared to control mice (38, 39). We observed that *db/db* mice rapidly gained weight (34.73 ± 4.996 g SD) compared to their heterozygous controls (19.30 ± 4.073 g SD) and developed hyperglycemia (293.0 ± 42.48 mg/dl SD, n = 4), while *db/+* mice maintained normal blood sugar (166 ± 69.16 mg/dl SD, n = 3). After isolation, islets from the mice were treated with control DMSO or Dan for 16 h before [Ca^2+^]_cyto_ measurements were carried out. As shown in Figure 11*A*, islets from *db/+* mice treated with vehicle lacked oscillations in 5 mM glucose, and as expected, exhibited normal oscillations in response to 11 mM glucose; these responses were unaffected by pretreatment with Dan. In contrast, *db/db* mice exhibited oscillations in 5 mM glucose and plateaus in 11 mM glucose. In these islets, the inclusion of Dan abolished the oscillations and decreased the percentage of oscillating islets observed from ∼70% to 25% in 5 mM glucose (Fig. 11*B*), without affecting the plateaus seen in 11 mM glucose (Fig. 11*A*).

## Discussion

We previously reported that [Ca^2+^]_cyto_ oscillations and increased insulin secretion observed in ER-stressed beta cells in subthreshold glucose are triggered by ER Ca^2+^ reduction and SOCE activation (20). SOCE activation induces a Ca^2+^ current that we believe shifts the cytosolic Ca^2+^ oscillatory threshold to the left, so that membrane potential as well as cytosolic Ca^2+^ now oscillate even in sub-threshold glucose levels (5 mM glucose) (20, 40). However, how ER stress reduces ER Ca^2+^ has remained unclear. Therefore, in the present study, we attempted to differentiate possible roles for RyRs and IP3Rs in ER Ca^2+^ reduction and its downstream effects on beta-cell Ca^2+^ homeostasis. Taken together, the data support the hypothesis that SOCE activation occurs secondary to RyR1-mediated ER Ca^2+^ reduction.

Although TM reduced [Ca^2+^]_ER_ in 5 mM glucose, blocking SERCA with CPA further decreased basal [Ca^2+^]_ER_ (Fig. 2*F*), but to a lesser degree compared to control (Fig. 2*G*) as [Ca^2+^]_ER_ was lower in TM-treated beta cells (Fig. 2, *B* and *C*). While blocking IP3Rs with XeC also prevented a loss in [Ca^2+^]_ER_ in TM-treated islets (Fig. 2*B*), the IP3R blocker XeC failed to inhibit TM-induced subthreshold [Ca^2+^]_cyto_ oscillations (Fig. 3*C*) or beta-cell apoptosis (Fig. 10*A*). On the other hand, blocking RyR1 with Dan suppressed the TM-induced subthreshold [Ca^2+^]_cyto_ oscillations (Fig. 3*D*) and beta-cell apoptosis (Fig. 10*B*) in addition to preventing the reduction in [Ca^2+^]_ER_ induced by TM (Fig. 2*C*). Similar results were reported in a study of Dan as a potential treatment for Wolfram syndrome, a rare autosomal recessive disorder that is associated with childhood-onset diabetes mellitus as well as sensory and neurological deficits (41). The causative genes for Wolfram syndrome are *WFS1* and *WFS2*, which encode ER-resident proteins. Loss of *WFS1* or *WFS2* has been shown to cause ER stress and decreased [Ca^2+^]_ER_ in neurons (42). Lu *et al.* found that knocking down WFS1 in INS-1 832/13 cells or NSC34 cells increased [Ca^2+^]_cyto_ and induced cell death, and importantly, these changes were suppressed by Dan (10 μM, 24 h). Moreover, Dan inclusion (10 μM, 48 h) also inhibited TG-induced cell death in neural progenitor cells derived from the induced pluripotent stem cells of a Wolfram syndrome patient (41).

In addition to inducing ER stress with chemical agents such as TM (Fig. 3) or TG (Fig. 9*C*), we also exposed islets to glucotoxic (25 mM, 16 h) or GLT conditions (400 μM palmitate, 16.7 mM glucose, 18 h) to mimic the milieu beta cells are exposed to in T2D; these treatments also trigger ER stress (Fig. S1*A*) (33). We examined the effect of Dan on [Ca^2+^]_cyto_ in islets that were exposed to high glucose or GLT and found that Dan suppressed subthreshold [Ca^2+^]_cyto_ oscillations seen in response to high glucose or GLT (Fig. 9, *A*, *B*, *D* and *E*). In addition, we observed that subthreshold [Ca^2+^]_cyto_ oscillations were prevented by Dan in islets from *db/db* mice (Fig. 11). These data taken together further support a role for RyR1 in ER stress-mediated Ca^2+^ dysfunction and show that our results are not limited to findings obtained using chemical stressors solely.

Yamamoto *et al.* showed that blocking RyRs with ryanodine prevented TM-induced sub-threshold [Ca^2+^] oscillations and cell apoptosis and suggested RyR2 to be the mediator due to its abundance in beta cells (18). As ryanodine is not selective for any of the RyR isoforms, and RyR2 is the dominant isoform of RyRs in beta cells (certainly by mass (18)), we silenced RyR1 and RyR2 individually in INS-1 832/13 cells and found that silencing RyR1 suppressed TM-induced subthreshold [Ca^2+^]_cyto_ oscillations, but silencing RyR2 did not (Figs. 4 and 5). Moreover, silencing RyR1 and RyR2 individually or together failed to prevent UPR activation (Fig. 7, *A* and *B*), in accordance with results we obtained using Dan or ryanodine (Fig. 6, *D*–*F* and Fig. 7, *C* and *D*). These data taken collectively suggest that [Ca^2+^]_ER_ reduction in our system may be downstream of UPR activation by TM, although we are not ruling out other mechanisms, such as reducing SERCA activity (43, 44).

Our finding that blocking IP3Rs or RyR1, while having similar suppressive effects on ER Ca^2+^ depletion to ER stressors nonetheless had differential effects on beta-cell apoptosis and SOCE activation/subthreshold [Ca^2+^]_cyto_ oscillations seems paradoxical, if ER Ca^2+^ depletion is indeed causative. However, it may be that the two classes of ER Ca^2+^ channels have different spatial distributions such that RyRs due to their localization within the cell are capable of selectively activating ER Ca^2+^ release in the vicinity of the SOCE mechanism. Thus, these receptors may not be uniformly distributed within the cell but selectively and dynamically organized into specific subcellular domains (45, 46). Stromal interaction molecule 1 (STIM1) and Ca^2+^ release-activated Ca^2+^ channel protein 1 (ORAI1) are the molecular subunits of SOCE channels. STIM1 resides in the ER membrane where it monitors the level of Ca^2+^ in the ER lumen (47, 48). In TM- or TG-treated beta cells, RyR1 might be in closer proximity to STIM1 than IP3Rs or RyR2, such that portions of the ER that are close to the plasma membrane preferentially contain RyR1 and hence that these ER subdomains empty more extensively, activating STIM1 and thus more readily coupling to ORAI1 to mediate Ca^2+^ entry from the extracellular space into the cytosol (SOCE) (40, 41). In contrast, in high glucose or GLT-treated beta cells, IP3Rs might also localize closer to STIM1/ORAI1 such that both receptors mediate ER Ca^2+^ reduction and SOCE activation equally well, so that XeC could in this case also inhibit subthreshold Ca^2+^ oscillations, although less efficaciously than Dan (Fig. 9).

For this scenario to hold, however, we posit that while D4ER indeed measures global ER Ca^2+^, individual portions of the ER may be too small or poorly sampled by D4ER to resolve differences in these very small and highly localized subdomains. For example, there may be portions of the ER Ca^2+^ reticular network where RyR1 is dominantly expressed over RyR2 or IP3Rs and which is in closer proximity to SOCE. Therefore, although inhibiting IP3Rs prevented a global ER Ca^2+^ loss in response to TM, SOCE activation was not inhibited because ER Ca^2+^ was still low in a STIM1 localized subcompartment of the ER. On the other hand, RyR1, despite its relatively lower abundance in beta-cells, is selectively localized near STIM1 and ORAI1, making the SOCE compartments very sensitive to Ca^2+^ release by RyR1 localized to this ER Ca^2+^ pool. Resolving the spatial localization of these molecules within the ER will require higher resolution imaging approaches than that used here, ones that can quantitatively and accurately assay the subcellular localization of RyR1, IP3Rs, STIM1, and ORAI1 under various ER stress conditions.

In summary, the present report demonstrates that RyR1 is a critical player in ER stress-induced ER Ca^2+^ loss and downstream alterations in beta-cell function and viability. Combining these new data with existing knowledge of RyRs and IP3Rs suggests that RyR1 might be a potentially useful therapeutic target for treatment during the onset or progression of type 2 diabetes.

## Experimental procedures

### Materials

TM, CPA, TG, and XeC were obtained from Cayman Chemical, Ann Arbor, MI. Dan was from Sigma-Aldrich, St Louis, MO. Ryanodine (Ry) was from Abcam, Waltham, MA. RNeasy mini kit for RNA extraction was from Qiagen, Germantown, MD. Superscript RT II was from Invitrogen, Carlsbad, CA. SYBR Green PCR master mix was from Applied Biosystems, Bedford, MA. Primers for qRT-PCR were from Integrated DNA Technologies, Coralville, Iowa. Small interfering RNAs (siRNAs) were from ThermoFisher scientific, Waltham, MA. The catalog and lot numbers of these materials can be found in Table S1.

### Isolation of pancreatic islets

Pancreatic islets were isolated from male Swiss-Webster mice (3 months of age; 25–35 g) and female *db/db* mice (BKS.Cg-Dock7^m^+/+Lepr^db^/J) and female heterozygous mice at 5 to 7 weeks of age, as described (49). Animal procedures were approved by the Institutional Animal Care and Use Committee of the University of Michigan (protocol PRO11164). Islets were cultured in RPMI 1640 medium containing 11 mM glucose, 10% fetal bovine serum (FBS), 10 mM HEPES, 1% penicillin/streptomycin and 1% sodium pyruvate. Sodium palmitate (from Dr Scott Soleimpour’s lab)/BSA coupling was generated as described (50).

### Cell culture and transfection

INS-1 832/13 cells were grown in standard RPMI 1640 medium as described above in 6-well plates at 37 °C in a 5% CO_2_ humidified atmosphere and used for experiments after reaching ∼70% confluency. INS-1 832/13 cells were transfected with *RyR1*-specific siRNA, *RyR2*-specific siRNA, or negative control siRNA using lipofectamine RNAiMAX reagent as described in the manufacturer’s protocol (Invitrogen). Transfection was assessed by qPCR.

### Quantitative PCR

Total RNA was extracted from INS-1 832/13 cells and reverse-transcribed to cDNA as described (20). qPCR was carried out using the primers listed in Table S2, and data were analyzed as described in (20) with expression presented relative to endogenous controls, HPRT1.

### [Ca^2+^]_cyto_ imaging

Islets were loaded with Fura-2/AM (2.5 μM) for 45 min in RPMI medium including 5 mM glucose. Islets were then transferred to a 1 ml perfusion chamber containing imaging buffer for 6 min, followed by perfusion at ∼1 ml/min. Imaging buffer contained (in mM): 140 NaCl, 3 CaCl_2_, 5 KCl, 2 MgCl_2_, 10 HEPES, and 5 glucose. Similarly, INS-1 832/13 cells seeded on glass coverslips were loaded with Fura-2/AM (2.5 μM) for 30 min in standard RPMI 1640 medium containing 11 mM glucose. Coverslips containing INS-1 832/13 cells were transferred to the perfusion chamber and imaged in imaging buffer. Ratiometric Fura-2/AM imaging was performed using 340/380 nm excitation and collecting 502 nm emission, as previously described (49). Fluorescence ratios were acquired using Metafluor software (Molecular Devices) and plotted using GraphPad Prism (GraphPad Software). The catalog and lot numbers of these materials can be found in Table S1.

### [Ca^2+^]_ER_ imaging

[Ca^2+^]_ER_ was measured using an ER-localized FRET biosensor D4ER (51). The same system described above for Fura-2/AM imaging was employed but using 430 nm for excitation and 470/535 nm to obtain ratiometric emission. The imaging solution used contained (in mM): 140 NaCl, 3 CaCl_2_, 5 KCl, 2 MgCl_2_, 10 HEPES, 5 glucose, and 0.2 diazoxide (Dz). Dz was included to keep the K_ATP_ channel in its open state to prevent oscillatory Ca^2+^ activity and improve the signal/noise ratio and stability of the ER Ca^2+^ recordings. FRET ratios were acquired using Metafluor software, plotted using Prism, and mean values were calculated using Excel.

### Assays of apoptosis

INS-1 832/13 cells were harvested and prepared for sub-G1 apoptosis assay as described (20). The percentage of apoptosis was determined by calculating the percentage of cells present in the sub-G1 phase in the DNA content histogram using a flow cytometer housed in the Flow Cytometry Core of the University of Michigan.

### Statistical analysis

Data were expressed as means ± SD and analyzed using one sample *t* test (Prism) with hypothetical value set for 1.0 or student’s *t* test when comparing two groups as indicated in the figure legends. Differences between two or more groups were analyzed using ordinary one-way ANOVA or two-way ANOVA (Prism) as specified in the figure legends with post hoc multiple comparisons by Tukey’s procedure. Values of *p* < 0.05 were considered statistically significant.

## Data availability

All data are contained within the manuscript.

## Supporting information

This article contains supporting information.

## Conflict of interest

The authors declare that they have no conflicts of interest with the contents of this article.
