# Supplementary material for: ER stress increases expression of intracellular calcium channel RyR1 to modify Ca2+ homeostasis in pancreatic beta cells
Source: J Biol Chem. 2023 Jul 17;299(8):105065. doi: 10.1016/j.jbc.2023.105065 (PMC10448220; doi:10.1016/j.jbc.2023.105065)
Supplement: Supporting Information [file mmc1.pdf]

Figure S1 High glucose increased *CHOP* expression

INS-1 832/13 cells were cultured in control 11 mM glucose or high glucose (HG, 25 mM) for 16 hours. S1A: *CHOP*; S1B: *RyR1*; S1C: *RyR2*; S1D: *IP3R1* mRNA levels were measured. All values shown are means ± SD, ##, $p < 0.01$; $n = 4$ independent experiments, by one sample t-test with hypothetical value set for 1.0.

Table 1 The list of materials

Table 2 The list of qPCR primer sequences for rat
