# Supplementary material for: ER stress increases expression of intracellular calcium channel RyR1 to modify Ca2+ homeostasis in pancreatic beta cells
Source: J Biol Chem. 2023 Jul 17;299(8):105065. doi: 10.1016/j.jbc.2023.105065 (PMC10448220; doi:10.1016/j.jbc.2023.105065)
Supplement: Supporting Table S1 [file mmc2.pdf]

Table 1

Materials	Source	REF/CAT number	LOT number
Tunicamycin	Cayman chemical	11445	11089-65-9
Cyclopiazonic acid	Cayman chemical	11326	18172-33-3
Thapsigargin	Cayman chemical	10522	67526-95-8
Sodium palmitate	Millipore Sigma	P9767	408-35-5
BSA	Millipore Sigma	A1470	9048-46-8
Dantrolene	Sigma-Aldrich	D9175	066M4126V
Xestospongin C	Cayman chemical	64950	88903-69-9
Ryanodine	Abcam	Ab120083	APN14103-1-1
DMSO	Sigma-Aldrich	472301	SHBK4364
RNeasy mini kit	Qiagen	74104	56905247
SuperScript lll	Invitrogen	11752	2358356
SYBR Green PCR master mix	Applied Biosystems	4367659	2203622
RPMI 1640 medium	Life technologies	11875-093	2339171
Penicillin/streptomycin	Life technologies	15140122	N/A
Sodium pyruvate	Life technologies	11360-070	2428722
Fetal bovine serum (FBS)	R&D systems	S11150H	B22030
HEPES solution	Life technologies	15630-080	1897332
Lipofectamine RNAiMAX	Invitrogen	56532	2373385
DNase l	Invitrogen	18068	2245072
Fura-2/AM	EMD Millipore	344911	3732343
NaCl	Sigma-Aldrich	S9888	SLCJ3968
$CaCl_2$	Sigma-Aldrich	M9272	021M0107V
KCl	Sigma-Aldrich	P3911	SLBG1548V
$MgCl_2$	Sigma-Aldrich	M9272	021M0107V
HEPES powder	Sigma-Aldrich	H3375	SLCJ4491
Glucose	Sigma-Aldrich	G8270	SLBP4805Vb
EGTA	Sigma-Aldrich	E-4378	50H5624
Diazoxide	Sigma-Aldrich	D9035	BCCB0466
Propidium iodide	Sigma-Aldrich	P4864	25535-16-4
Gibco PBS	Fisher scientific	20012-027	2362348
UltraPure distilled water	Invitrogen by Life Technologies	10977-015	2186770
