# Supplementary material for: ER stress increases expression of intracellular calcium channel RyR1 to modify Ca2+ homeostasis in pancreatic beta cells
Source: J Biol Chem. 2023 Jul 17;299(8):105065. doi: 10.1016/j.jbc.2023.105065 (PMC10448220; doi:10.1016/j.jbc.2023.105065)
Supplement: Supporting Table S2 [file mmc3.pdf]

Table 2

| Gene | Forward | Reward |
|---|---|---|
| *RyR1* | CGTAGACAACAACAGGGCAC | AGATTTCTCCCACCATCCTGA |
| *RyR2* | ACGGCGACCATCCACAAAG | AAAGTCTGTTGCCAAATCCTTCT |
| *IP3R1* | CAACCGTTACTATGGAAACATC | TCAGCCAGGCTCATCTCAC |
| *IP3R2* | CGATGCCAGGATACGATGT | CACCCTTGAAGTACCGATT |
| *IP3R3* | AGGAGCTGGTGGACGTGAT | TGCTTGTTGTGCCTGGAAA |
| *Hprt1* | CTCATGGACTGATTATGGACAGGAC | GCAGGTCAGCAAAGAACTTATAGCC |
| Spliced *Xbp1* | CTGAGTCCGAATCAGGTGCAG | ATCCATGGGAAGATGTTCTGG |
| Total *Xbp1* | GAGCAGCAAGTGGTGGAT | TCTCAATCACAAGCCCATG |
| *ATF4* | CCTGACTCTGCTGCTTATATTACTCTAAC | ACTCCAGGTGGGTCATAAGGTTTG |
| *CHOP* | CCAGCAGAGGTCACAAGCAC | CGCACTGACCACTCTGTTTC |
