# Supplementary figures and images for: ER stress increases expression of intracellular calcium channel RyR1 to modify Ca2+ homeostasis in pancreatic beta cells
Source: J Biol Chem. 2023 Jul 17;299(8):105065. doi: 10.1016/j.jbc.2023.105065 (PMC10448220; doi:10.1016/j.jbc.2023.105065)

Figure S1

A)

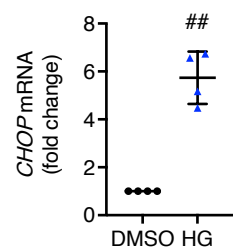

B)

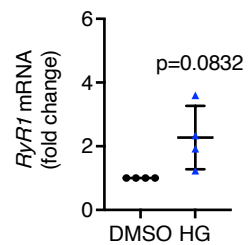

C)

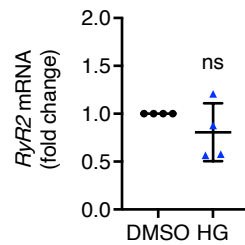

D)

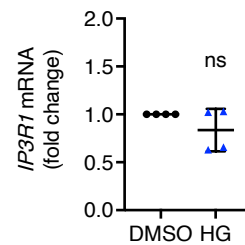

Supplement: Supporting Figure S1 [file mmc4.pdf]
